# Plasma oncology: Adjuvant therapy for head and neck cancer using cold atmospheric plasma

**DOI:** 10.3389/fonc.2022.994172

**Published:** 2022-09-29

**Authors:** Xuran Li, Xiaoqing Rui, Danni Li, Yanhong Wang, Fei Tan

**Affiliations:** ^1^ Shanghai Fourth People’s Hospital, and School of Medicine, Tongji University, Shanghai, China; ^2^ Shanghai East Hospital, Shanghai, China; ^3^ Department of Surgery, The Royal College of Surgeons in Ireland, Dublin, Ireland; ^4^ Department of Surgery, The Royal College of Surgeons of England, London, United Kingdom

**Keywords:** cold atmospheric plasma, plasma medicine, head and neck cancer, clinical application, plasma oncology

## Abstract

The worldwide incidence of head and neck cancer (HNC) exceeds half a million cases annually, and up to half of the patients with HNC present with advanced disease. Surgical resection remains the mainstay of treatment for many HNCs, although radiation therapy, chemotherapy, targeted therapy, and immunotherapy might contribute to individual patient’s treatment plan. Irrespective of which modality is chosen, disease prognosis remains suboptimal, especially for higher staging tumors. Cold atmospheric plasma (CAP) has recently demonstrated a substantial anti-tumor effect. After a thorough literature search, we provide a comprehensive review depicting the oncological potential of CAP in HNC treatment. We discovered that CAP applies to almost all categories of HNC, including upper aerodigestive tract cancers, head and neck glandular cancers and skin cancers. In addition, CAP is truly versatile, as it can be applied not only directly for superficial or luminal tumors but also indirectly for deep solid organ tumors. Most importantly, CAP can work collaboratively with existing clinical oncotherapies with synergistic effect. After our attempts to elaborate the conceivable molecular mechanism of CAP’s anti-neoplastic effect for HNC, we provide a brief synopsis of recent clinical and preclinical trials emphasizing CAP’s applicability in head and neck oncology. In conclusion, we have enunciated our vision of plasma oncology using CAP for near future HNC treatment.

## 1 Introduction

### 1.1 Head and neck cancer and its treatment

Head and neck cancer (HNC) was the seventh most common cancer worldwide and comprise of tumors affecting the upper aerodigestive tract (1, 2). HNC encompasses three categories: the upper aerodigestive tract cancers (oral cavity, nasal cavity, paranasal sinuses, pharynx, and larynx), head and neck glandular cancers (thyroid gland and salivary gland), and head and neck skin cancers. Many head and neck cancers arise from mucosal epithelial cells in the oral cavity, pharynx, and larynx, and the most common histological type is squamous cell carcinoma (SCC) (3). The mainstay treatment paradigm for HNC includes surgery, radiotherapy, and chemotherapy including platinums, taxanes, antimetabolic agents, and DNA damaging agents (4–6) (**Table 1**). In addition, the immune checkpoint inhibitors, pembrolizumab and nivolumab, have been approved by the FDA for the treatment of cisplatin-refractory recurrent or metastatic head and neck squamous cell carcinoma (HNSCC) (15, 16). Despite receiving these therapies, more than 65% of patients with advanced HNSCC have recurrence or metastasis, and only half of the patients remain alive after five years (17). In addition, advanced HNC is often accompanied by complications and treatment side effects, such as severe infection and persistent tumor ulcers, which further reduce the patients’ quality of life. Therefore, there is a great need to explore more treatment modalities for controlling HNC.

**Table 1:** Commonly used chemotherapeutic drugs for head and neck cancer.

Drug type	Subtype	Commercial drugs	Combined with CAP	Refs
Chemotherapy	Platinum	Cisplatin	Y	(7)
		Carboplatin	NA	(8)
	Taxane	Paclitaxel	NA	(6)
		Docetaxel	NA	(9)
	Antimetabolic agents	Fluorouracil	NA	(10)
		Methotrexate	NA	(11)
		Hydroxyurea	NA	(12)
	DNA damaging agents	Bleomycin sulfate	NA	(4)
Immunotherapy	PD-1 antibodies	Nivolumab	Y	(13)
		Pembrolizumab	NA	(15)
Targeted therapy	EGFR antibodies	Cetuximab	Y	(14)

(EGFR, epidermal growth factor receptor; PD-1, programmed cell death protein 1; Y, yes; NA, not applicable).

### 1.2 Cold atmospheric plasma

Plasma, the fourth state of matter, has been widely used in industry in the past few decades. Cold atmospheric plasma (CAP) is a partially ionized gas that is generated under atmospheric pressure at room temperature (18). It has been adopted in the medical field because of its host-friendly low temperature. The cold atmospheric plasma can be generated by various technologies. Piezoelectric direct discharge (PDD) is a new technology which is based on the resonant piezoelectric transformer (RPT) principle (19). Compared to the traditional corona discharge (CD) and dielectric barrier discharge (DBD) technology, PDD is more versatile using different excitation structures. The Piezobrush^®^ compact handheld plasma devices, PZ2 and PZ3, are based on PDD technology. In order to improve the potential of CAP, argon (Ar), helium (He), oxygen (O_2_), nitrogen (N_2_), air, and two or more of them can be utilized to generate CAP.

### 1.3 CAP’s anti-neoplastic effect

**Figure 1:**
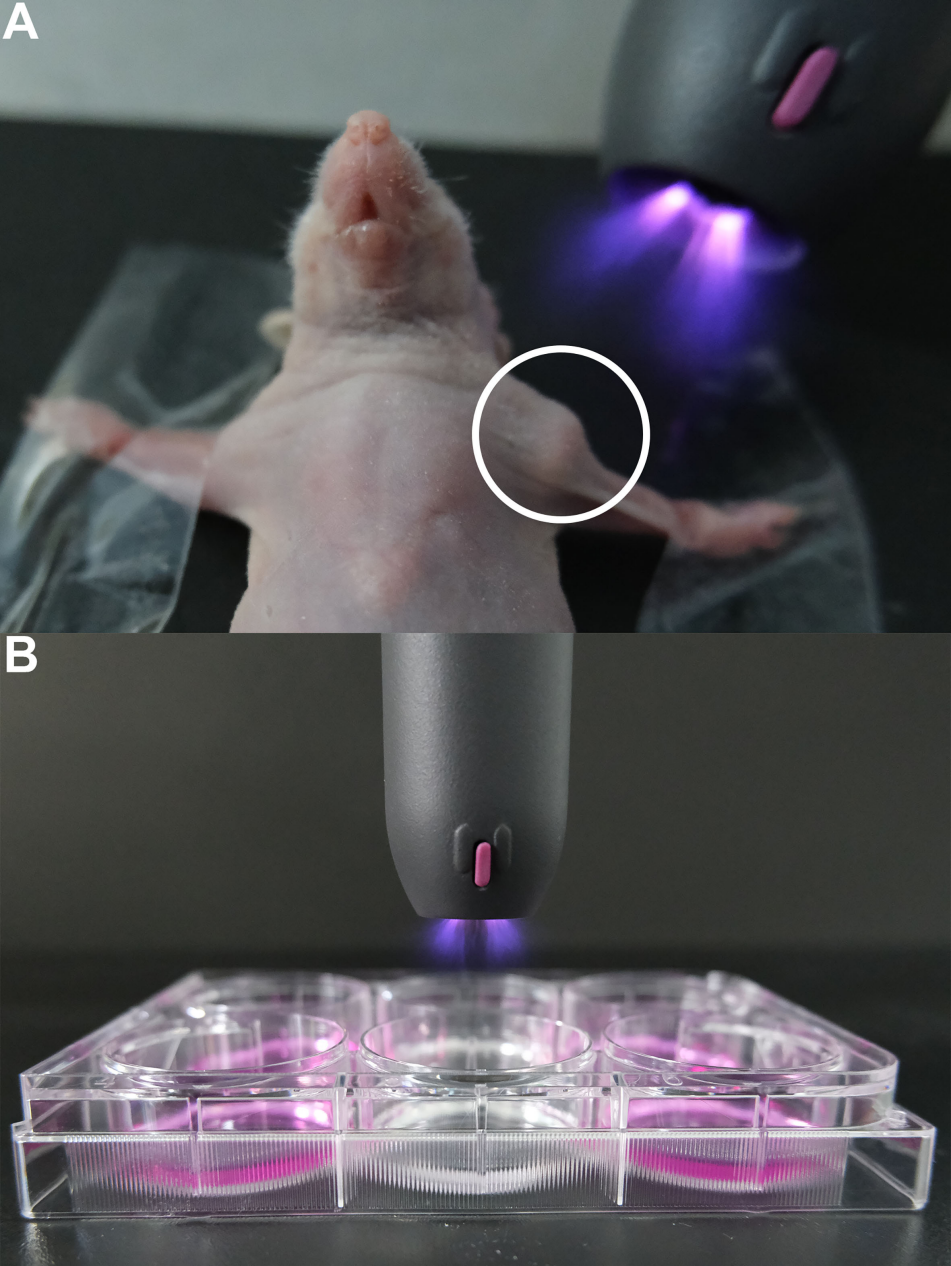
Examples of the direct and indirect applications of CAP in HNC plasma oncology. The portable Piezobrush CAP system is used for illustration. **(A)** Direct treatment of human tumor CDX model using CAP (the circle highlights the implanted tumor in an immunodeficient mouse). **(B)** Indirect treatment of HNC cell culture in various media. (CAP, cold atmospheric plasma; CDX, cell line-derived xenograft; HNC, head and neck cancer).

The potential contributors by which CAP inhibits cancer development include long-lived and short-lived reactive species, charged particles, pressure gradients, and changes in electromagnetic fields. CAP produces a large number of reactive oxygen species (ROS) and reactive nitrogen species (RNS), which impact tumor cells by increasing their oxidative stress. The RONS could be classified into interconverting long-lived and short-lived reactive species. The short-lived particles include singlet oxygen (^1^O_2_), hydroxyl radicals (^•^OH), superoxide anions (O_2_^-^), and peroxynitrite (ONOO^-^). The ^1^O_2_ is a highly active molecule that is involved in the apoptosis process and produces cytotoxic chemicals. The O_2_^-^ is also highly reactive and can increase the oxidative stress that the tumor cells are exposed to. The hydroxyl radicals were extremely reactive oxidizing species, which contribute to the oxidation of lipid, nuclide and protein, resulting in cell death. On the other hand, the long-lived particles compromise hydrogen peroxide (H_2_O_2_), nitrite (HNO_2_/NO_2_^-^), and nitrate (HNO_3_/NO_3_^-^). The effect of the long-lived RONS generated by CAP has been described in the plasma-activated medium (PAM) treatment (**Figure 1**). The H_2_O_2_ entered tumor cells as a secondary messenger for triggering signaling cascades. The NO_2_^-^ and NO_3_^-^ enhance the anti-tumor effect of CAP by generating short-lived ONOO^-^. In addition, the oxidative stress caused by CAP also modifies the amino acids of proteins, contributing to structure distortion and dysfunction of these proteins (20).

Physical factors are also essential components of CAP. One recent study suggested that magnetic field can induce the activation of cancer cells, rendering them more susceptible to ROS (21). In addition, other physical species produced by CAP include highly energetic photons in the ultraviolet. They can impact energy transport and break chemical bonds in cancer cells (22). During this process, some proteins and DNA of cells may absorb this energy and turn abnormal. These highly energetic photons can even induce photolysis in liquid, thus producing ^•^OH radicals (23).

### 1.4 Potential of CAP in HNC treatment

**Figure 2:**
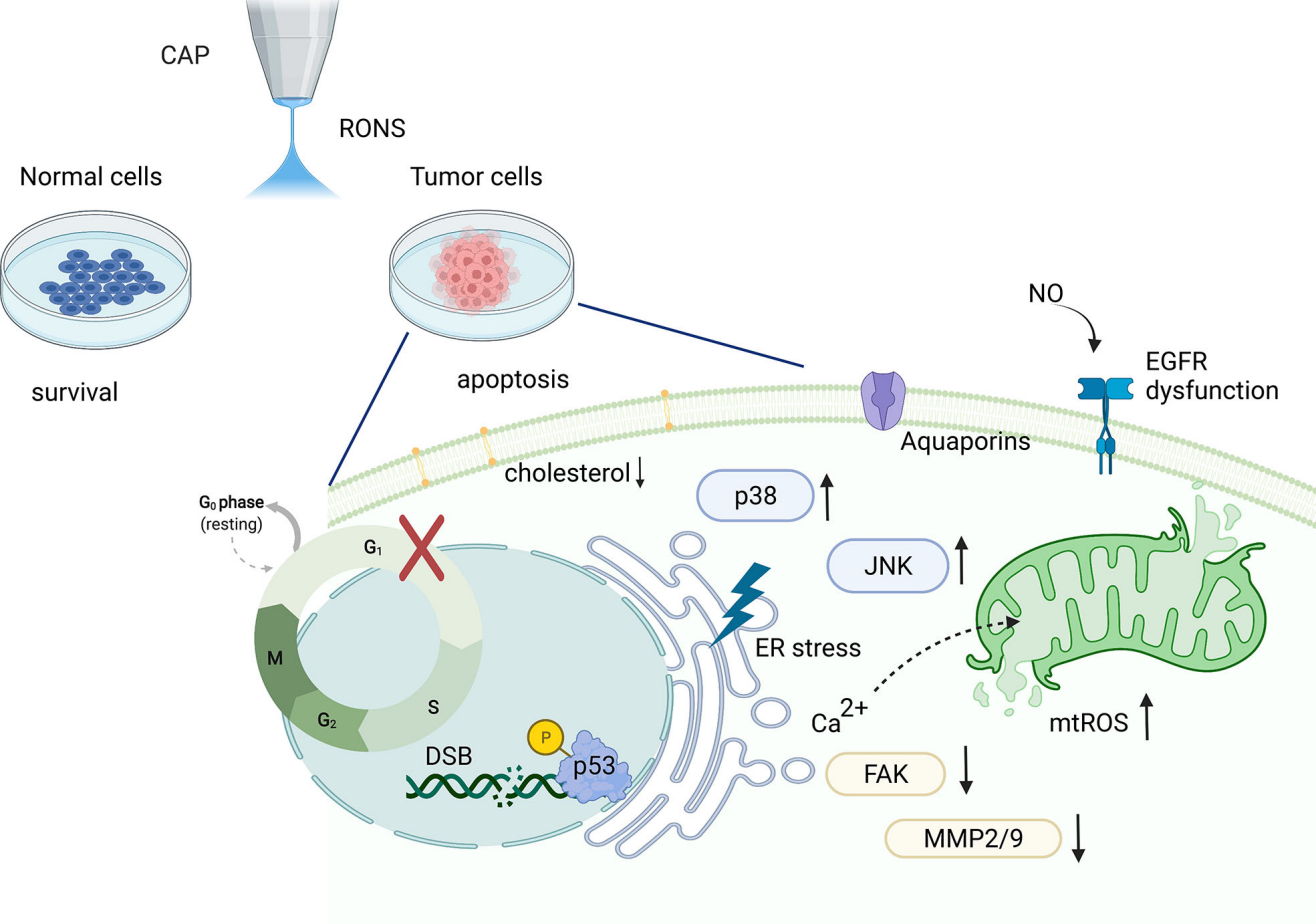
Schematics of CAP treatment of HNSCC cells and the potential underlying molecular mechanism. CAP demonstrates selectivity to cancer cells sparing adjacent healthy cells. In addition, CAP treatment induces a series of DNA damage and mitochondrial dysfunction in cancer cells. Moreover, CAP activates several tumor progression-associated signalling pathways, such as the p53 and MAPK signalling pathways. (CAP, cold atmospheric plasma; DSB, DNA double-stranded breakage; EGFR, epidermal growth factor receptor; ER, endoplasmic reticulum; FAK, focal adhesion kinase; MAPK, mitogen-activated protein kinases; MMP, matrix metalloproteinase; mtROS, mitochondrial reactive oxygen species; NO, nitric oxide; JNK, Jun N-terminal kinase; RONS, reactive oxygen and nitrogen species).

Recently, the potential application of CAP in HNC treatment has increasingly raised attention. Unlike other applications of CAP, such as antimicrobial management and promoting wound healing (24), the applications of CAP in HNC treatment aim at microscopically killing the tumor cells and macroscopically alleviating the tumor-associated symptoms. In order to elucidate the underlying mechanism and demonstrate the therapeutic effect of CAP in HNC treatment, we designed this review with twofold implications, bridging the gap between plasma biotechnology (**Table 2**) and clinical oncology (**Table 3**). Horizontally, we discussed HNCs that apply to CAP treatment, based on their anatomical subsites. Vertically, we interpreted the molecular mechanisms of CAP therapy for each subtype of HNCs (**Figure 2**). Finally, we provided a summary of the latest examples of successful preclinical and clinical trials using adjuvant CAP therapy.

**Table 2:** Overview of studies on CAP treatment in head and neck cancer.

Category of cancer	Subtype	Test model	Synopsis	Refs
**Head and neck upper aerodigestive tract cancers**	SCC	JHU-022, JHU-028, JHU-029 and SCC25 cells	CAP selectively eliminates HNSCC cell lines through non-apoptotic mechanisms, with minimal effect on normal oral epithelial cell lines	(25)
		HSC-2, SCC-15 cells	CAP induces EGFR dysfunction in EGFR-overexpressing oral SCC *via* NO radicals	(26)
		MSKQLL1, SCC1483 cells	among 3 gases, N_2_ CAP inhibited cell migration and invasion most potently *via* decreased FAK & MMP	(27)
		FaDu, SNU1041and SNU899 cells, mice	CAP induces apoptosis of HNC cells through a mechanism involving MAPK-dependent mitochondrial ROS	(28)
		SNU1041, SNU1076 and SCC25 cells	PAM induces HNC cell death *via* the upregulation of ATF4/CHOP activity by damaging mitochondria through excessive mtROS accumulation	(29)
		MSK QLL1, SCC1483, SCC15 and SCC25 cells	CAP induced OSCC cell apoptosis through a mechanism involving DNA damage and triggering of sub-G1 arrest *via* the ATM/p53 pathway	(30)
		FaDu, SCC15, SCC-QLL1, SCC1483 andHN6 cells	PAM inhibits tumor progression by increasing the MUL1 level and reducing p-AKT level	(31)
		KYSE-30 cells	CAP induces genotoxicity and cytotoxicity in cancer cells	(32)
		SCC25 cells	the killing efficiency of CAP in the presence of EGFR antibody conjugated GNP is amplified about 18 times	(33)
		SCC25 cells	PD-L1 antibody + GNP + CAP significantly increased the number of dead cancer cells	(13)
		MSKQLL1, SCCQLL1, HN6, SCC25, SCC15, Cal27 and SCC1483 cells	A combination of CAP and cetuximab shows inhibited invasion and migration *via* NF-κB signaling in cetuximab-resistant OSCC cells	(14)
		SCC-15 cells	The synergy of cisplatin & CAP reduces the required dosage of chemotherapy	(34)
**Head and neck glandular cancers**	thyroid gland cancer	SNU80 cells	The altered antioxidant system stimulates CAP-induced cell death	(35)
		BHP10-3 andTPC1 cells	CAP inhibits cell invasion *via* cytoskeletal modulation and altered MMP-2/-9/uPA activity	(36)
		BCPAP, HTh7, KTC2, 8505C, and FRO-Luc cells	activation of EGR1/GADD45α by CAP mediates thyroid cancer cell death	(37)
		HTH83, U-HTH 7, and SW1763 cells	CAP induces apoptosis in anaplastic thyroid cancer cell lines *via* the JNK signaling pathway and p38 MAPK-dependent ROS	(38)
	parotid gland cancer	HN9 cells	CAP induces apoptosis in HN9 cells	(28)
**Head and neck skin cancers**	melanoma	A375 cells	combined therapy using PpIX-loaded polymersome-mediated PDT and CAP	(39)
		G-361 cells, mice	CAP & SN synergistically inhibit mitochondrial function *via* the HGF/c-MET signalling pathway	(40)
		G-361 cells	CAP & SN activate autophagy by activating PI3K/mTOR and EGFR pathways	(41)
		L929 cells, mice	both CAP monotherapy and combination with chemotherapy significantly decreased tumor growth	(42)
		Mel Juso cell line	The synergy between CAP-induced RONS and acidic conditions promotes anti-cancer effects	(43)
		A375 and A875 cells	CAP increases Sestrin2 expression and further activates Fas *via* the MAPK signaling pathway	(44)
		SK-Mel-147 cells	CAP inhibits migration and disorganizes the actin cytoskeleton through multiple signaling pathways, using transcriptomic analysis	(45)
		Mel Im cell line	CAP changes the amino acid composition of the cell culture medium and affects the anti-tumor mechanism	(46)
	BCC	TE354T cells	PAM induces apoptosis *via* MAPK signalling pathway	(47)

(ATF4, activating transcription factor 4; ATM, ataxia telangiectasia-mutated gene; BCC, basal cell carcinoma; CAP, cold atmospheric plasma; CHOP, C/EBP homologous protein; c-MET, c-mesenchymal-epithelial transition factor; ECT, electrochemotherapy; EGFR, epidermal growth factor receptor; EGR1, early growth response 1; FAK, focal adhesion kinase; GNP, gold nanoparticles; HGF, hepatocyte growth factor; HNC, head and neck cancer; HNSCC, head and neck squamous cell carcinoma; JNK, c- Jun N-terminal kinase; MAPK, mitogen-activated protein kinase; MCT, multi cellular tumor spheroids; MMP, matrix metalloproteinase; mtROS, mitochondrial reactive oxygen species; mTOR, mammalian target of rapamycin; MUL1, mitochondrial E3 ubiquitin protein ligase1; N_2_, nitrogen; NO, nitric oxide; O_2_, oxygen; OSCC, oral squamous cell carcinoma; PAM, plasma-activated medium; PDT, photodynamic therapy; PI3K, phosphatidylinositol 3 kinase; PpIX, protoporphyrin IX; PD-L1, programmed death-ligand 1; ROS, reactive oxygen species; RNS, reactive nitrogen species; SCC, squamous cell carcinoma; SMD, surface micro discharging; SN, silymarin).

**Table 3:** Recent preclinical and clinical trials of CAP treatment in head and neck cancer.

Category of cancer	No. of pts	Tumor location & subunits	Staging	Symptomatic improvement	Tumor suppression	Side effects	Refs
**Head and neck upper aerodigestive tract cancers** **(SCC and adenocarcinoma)**	12	floor of mouth (n = 5), hypopharynx (n = 1), tonguebase (n= 1), upper jaw (n= 2), lower jaw (n= 3)	T3/4	decreased microbial load, odor, and pain (6/12)	partial remission (4/12)	bad taste, pain, exhaustion, edema, and bleeding	(48)
	21	head and neck	T3/4	vascular stimulation or a contraction of tumor ulceration (4/12)	increased cell apoptosis in CAP-treated tissues	bad taste, pain, sialorrhea, exhaustion, edema, and bleeding	(49)
	2	neck	T3/4	improved microcirculation (2/2) and postoperative wound healing (2/2)	N/A	N/A	(50)
	6	oropharynx	T4	reduced odor and pain (5/6)	partial remission (2/6)	pain, sialorrhea, dry mouth, exhaustion, edema, and bleeding	(51)
	10	mouth (n = 5), cheek (n = 2), tongue (n = 1), retromolar region (n = 1), maxillary sinus (n = 1, adenocarcinoma)	T4	N/A	increased cell apoptosis in CAP-treated tissues	N/A	(52)
**Head and neck skin cancers** **(melanoma)**	6	metastatic cutaneous melanoma	T4	decreased secretion of inflammatory cytokines (1/3)	increased cell apoptosis in CAP-treated tissues	N/A	(53)

(CAP, cold atmospheric plasma; pts, patients; SCC, squamous cell carcinoma; N/A, Not available).

## 2 Methods

### 2.1 Literature review process

The PubMed database, Web of Science and Google Scholar were used to search the scientific literature for current researches related to CAP and HNC. The search terms included “head and neck neoplasm OR HNSCC OR HNC” and “cold atmospheric plasma”, “cold plasma”, “non-thermal atmospheric plasma”, “plasma medicine”. The final search included CAP-related articles published between 2011 and 2022. These include *in vitro* studies, preclinical studies, and clinical studies.

## 3 CAP’s treatment for head and neck upper aerodigestive tract cancers

The upper aerodigestive tract (ADT) consists of several subsites in the head and neck, serving respiratory and swallowing functions. These include the oral cavity, nasal cavity, paranasal sinuses, pharynx, and larynx. Ninety percent of the head and neck ADT malignant tumors are histologically squamous cell carcinoma (SCC) (54). The incidence of HNSCC is approximately 600,000 cases per year worldwide (55, 56).

### 3.1 CAP selectively eliminates cancer cells

The oncotherapy using CAP demonstrates selectivity to cancer cells over adjacent healthy cells. Yan et al. conducted a study comparing cancer cells with normal cells and found that CAP showed strong selectivity in most of the chosen cancer cell lines (57). There are multiple reasons for the selectivity of CAP treatment in HNSCC.

#### 3.1.1 Cell cycle factor for CAP’s selectivity

CAP’s selectivity to HNSCC cells may be related to cell cycle manipulation. It is well known that tumor cells proliferate rapidly, meaning a faster progression of the cell cycle with a high proportion of cells in the S phase. This may render cancer cells more vulnerable to plasma treatment (58). Compared to tumor cells, normal cells at the end of differentiation are rarely in the phase of division. Although data suggested that CAP treatment affects all phases of the cell cycle, the response of cells to CAP treatment varies depending on the phase distribution of the cell cycle. Interestingly, tracer assay and γH2A.X expression study have shown that cells in the S phase were more sensitive to CAP treatment.

#### 3.1.2 Cell membrane factor for CAP’s selectivity

There are many differences between tumor cells and normal cells in terms of cell membrane structure. The first difference in the cell membrane is the expression of aquaporin, a protein which is usually more abundant in tumor cells. These proteins function as a selective filter which could facilitate the transportation of small molecules, such as CO_2_, NO, and reactive oxygen species (ROS). Recent experiments have shown that increased expression of aquaporin could lead to increased concentration of intracellular ROS concentration, which contributes to the high level of oxidative stress (59). In addition, reduced cholesterol content in tumor cells renders the cell membrane incapable of providing a barrier against the entry of reactive species, such as H_2_O_2_ (60). Considering the high levels of preexisting ROS in cancer cells, additional ROS introduced by CAP could lead to a tipping of the balance with detrimental results.

#### 3.1.3 Cellular receptor factor for CAP’s selectivity

The epidermal growth factor receptor (EGFR) plays a vital role in the upper ADT mucosal cells and regulates the cell cycle, cell proliferation, and transformation. It is well known that EGFR is overexpressed in HNSCC cells. Lee et al. reported that the selective killing effect of CAP in oral SCC is associated with NO-induced dysfunction of EGFR (26). Moreover, pretreatment using NO scavenger rescued the dysfunction of the EGFR and the following killing effect.

Irrespective of the contributing factors mentioned above, CAP application could still selectively damage HNSCC cell lines with little effect on normal oral cell lines, in a non-apoptosis-inducing way. Guerrero-preston et al. applied CAP treatment on four HNSCC cell lines (JHU-029, JHU-028, JHU-022, SCC25) and two normal oral mucosa epithelial cell lines (OKF6 and NOKsi) (25). Due to the short duration of CAP treatment, significant apoptosis was not observed in either type of cells. They revealed that CAP selectively impaired HNSCC cell viability in a dose-dependent manner by MTT assays, and the viability of normal cells remained virtually unchanged.

### 3.2 CAP affects major cellular processes during cancer progression

In general, CAP reduces cell viability, inhibits cell migration, and induces apoptosis in tumor cells (61). There are several underlying molecular mechanisms that may lead to the above changes in tumor cell behavior. These include DNA damage, mitochondrial dysfunction, and other processes.

#### 3.2.1 DNA damage caused by CAP treatment

CAP causes a series of DNA damage in HNSCC cells. The main components of CAP are not only RONS but also charged particles, UV radiation, and electromagnetic fields (62). Previous studies have proven that all these components are associated with DNA damage (63–65). Chang et al. uncovered that the CAP treatment induced DNA double-strand breakage (DSB) and triggered sub-G1 arrest in oral SCC (OSCC) cells (30). Although the cell cycle can be arrested to allow for the repair of DNA damage, but if the damage is overwhelming and beyond repair, apoptosis will be induced. Large-scale image analysis has found that S phase-gathering cells were more susceptible to DNA damage than either G1 or G2 phase-gathering cells. Tumor cells happen to be mostly in the S phase due to their dividing nature. These results confirm that CAP induces DNA damage, particularly in cancer cells (32, 66).

#### 3.2.2 Mitochondrial dysfunction caused by CAP treatment

HNSCC cells exhibit reduced mitochondrial function and elevated numbers of disrupted mitochondria after CAP treatment. Specifically, an RNA sequencing analysis revealed that the mitochondrial oxidative phosphorylation-related genes tended to be down-regulated after CAP irradiation (29). Further experiments not only confirmed the accumulation of mitochondrial reactive oxygen species (mtROS) but also revealed that this was associated with ATF4/CHOP regulation using transcriptomic analysis. In addition, an increased level of RONS induced by CAP triggered endoplasmic reticulum stress, leading to a calcium influx into mitochondria. As a result, the excess calcium overwhelms the oxidative phosphorylation of mitochondria, decreases mitochondrial membrane potential, and induces mitochondria-related apoptosis.

### 3.3 CAP activates various signaling pathways in cancer cells

Several tumor progression-associated signaling pathways are activated during CAP treatment. These include, but are not limited to, p53, mitogen-activated protein kinases (MAPK), and other signaling pathways, depending on the mode of plasma treatment.

#### 3.3.1 The p53 signaling pathway is activated after CAP treatment

Under normal circumstances, p53 remains inactivated with a low expression level. When there is DNA damage, it could rapidly activate p53 through upregulation of the ATM gene (67). The activated p53 triggers its downstream effectors, which regulate cell cycle checkpoint and apoptosis (68). Similarly, the ATM/p53 signaling pathway plays an important role in the CAP-induced apoptosis in OSCC. After CAP treatment, the DNA damage triggered the activation of ATM, which led to the phosphorylation of p53. If the DNA damage is severe and cannot be repaired, the Ser 46 of p53 could be phosphorylated by ATM, and apoptosis ensued (30). In addition, indirect treatment using PAM also exerted similar anti-tumor effects to direct CAP treatment. Agene expression analysis showed that p53 pathway-related genes were also highly activated in OSCC cells treated by PAM (69).

#### 3.3.2 The MAPK signaling pathway is activated after CAP treatment

The MAPK signaling pathway is involved in cancer cell proliferation, inflammation, differentiation, migration and apoptosis (70). The main branching nodes of MAPK signaling pathway comprise of extracellular signal-related kinases (ERK1/2), Jun amino-terminal kinases (JNK), and p38 (71). Previous studies suggested that activation of ERK promotes cell proliferation, whereas that of JNK and p38 facilitates cell apoptosis (72). In FaDu cells (from hypopharyngeal SCC), CAP induced increased level of mtROS and apoptosis *via* the MAPK signaling pathway (28). In this study, increased levels of p-p38 and p-JNK were detected after CAP treatment. Blocking the activation of JNK and p38 using inhibitors rescued CAP-induced apoptosis and reduced mitochondrial damage.

#### 3.3.3 Other signaling pathways activated by CAP treatment

During direct CAP treatment of OSCC, tumor cell migration and invasion were inhibited through decreased focal adhesion kinase (FAK) expression and reduced matrix metalloproteinases (MMP2/9) activity (27). In comparison, indirect plasma treatment using PAM induced SCC cell detachment from multi-cellular tumor spheroids (MCTS) in a dose-dependent manner (73). After multiple treatments using PAM, inhibition of cell growth was observed in MCTS, and cell death in 2D cell cultures, respectively. Furthermore, PAM inhibited tumor progression by enhancing the expression of MUL1 and decreasing that of p-AKT in a xenograft tumor model (31).

### 3.4 CAP provides synergy with other oncotherapies

CAP, when combined with other oncotherapy, has the potential of manifesting synergistic effect. These trialed cancer treatments are mostly chemotherapy, targeted therapy, and immunotherapy.

#### 3.4.1 CAP combined with chemotherapy

Chemotherapy has been used as an anti-HNC treatment for a long time, but its success is not without side effects. Lee et al. reported that the combination of CAP treatment and chemotherapy proved synergistic (34). Their study discovered that this combinatorial treatment could increase the anti-tumor efficacy while reducing the required dosage of chemotherapy medication. This treatment mode greatly reduced the chance of normal cells being mistakenly killed by chemotherapy drugs.

#### 3.4.2 CAP combined with targeted therapy

As mentioned in section 3.1.3, EGFR is overexpressed or constitutively remains activated in HNC. Therefore, blocking EGFR has been regarded as one of the most promising targets for HNC treatment. CAP combined with EGFR antibody-conjugated gold nanoparticles (GNPs) showed high selectivity to tumor cells. It also amplified the anti-neoplastic effect about 18 times compared to plasma treatment in the absence of conjugates (33). Although EGFR has been one of the main molecular targets, only 10–20% of patients with HNSCC are sensitive to anti-EGFR therapy (74). Therefore, a strategy to overcome the resistance to EGFR treatment is clinically required. Cetuximab, a commonly used targeting agent, exerts its anti-tumor effect by competitively binding to EGFR. It is known that cetuximab with chemotherapy as a first-line treatment prolongs survival in HNCs. Interestingly, the combination of CAP and cetuximab showed inhibited invasion and migration in cetuximab-resistant OSCC cells (14). Further experiments revealed that this combination attenuated tumor invasion and migration *via* the NF-κB signaling pathway.

#### 3.4.3 CAP combined with immunotherapy

Programmed cell death protein 1 (PD-1) is an immune-inhibitory receptor expressed by T cells, and is involved in various physiological responses, such as infection, cancer and immune homeostasis. PD-L1, the ligand for PD1, is highly expressed in many cancers, including HNCs. Thus, blocking the PD-1/PD-L1 interaction by antibodies could inhibit tumor progression (75). In Park’s latest innovative study, the combination of CAP and immunotherapy showed a synergistic effect (13). They collectively applied CAP and GNP- conjugated PD-L1 antibodies in OSCC cells. This combinatorial treatment significantly increased the number of apoptotic cells when compared to the monotherapy groups.

## 4 CAP’s treatment for head and neck glandular cancers

### 4.1 Thyroid gland cancers

Located near the base of the neck, the thyroid is a large endocrine gland that produces and releases thyroid hormone. Thyroid cancer is the fifth most common cancer in women in the USA (76). Histologically, there are five types of thyroid cancer: papillary, follicular, medullary, anaplastic thyroid cancer, and thyroid lymphoma. Some subtype of thyroid cancer, especially anaplastic thyroid cancer, has a very poor prognosis and high mortality rate. Despite conventional treatments, such as surgery and radioactive iodine therapy, there is an urgent need for novel adjunctive therapies in cases of surgically challenging thyroid cancer.

#### 4.1.1 CAP’s selectivity for thyroid cancer cells

Just like CAP’s selectivity for SCC cells during HNC treatment (section 2.1), CAP is also selective for thyroid cancer cells. Kuashik et al. reported that the increased ROS in thyroid cancer cells, which were introduced by CAP, could contribute to cell apoptosis *via* alterations in GSH/GSSG ratio, NADP+/NADPH ratio, and total antioxidant activity (35). In addition, plasma decreased the metabolic viability and colony formation of thyroid cancer cells. These findings suggest a promising elimination of thyroid cancer cells by CAP irradiation while sparing normal healthy cells.

#### 4.1.2 Potential molecular mechanism of CAP’s selectivity

Further studies elucidated the molecular mechanism underlying CAP’s therapeutic effect on thyroid cancer. Jung et al. advised that CAP treatment in the form of PAM upregulated the gene expression of early growth response 1 (EGR1), which in turn increased the level of DNA damage-inducible 45α (GADD45A) through direct binding to its promoter (37). In their xenograft mouse tumor model, PAM inhibited thyroid cancer progression also by elevating EGR1 levels. In a different study, CAP inhibited the invasion and metastasis of thyroid papillary cancer cells by decreasing the expression of MMP2/MMP9 and uPA (36). This inhibitory effect was also accomplished by rearranging the cytoskeleton, which was regulated by the FAK/Src complex (36). These findings revealed that CAP could be a novel approach for locally invasive and metastatic thyroid cancers.

### 4.2 Salivary gland cancers

Major salivary glands, such as the parotid gland and submandibular gland, are both located in the head and neck region. Salivary gland cancer constitutes only 3% of all HNCs, and most salivary gland tumors arise in the parotid gland (77). Recent studies have shown that parotid gland carcinoma cells were also sensitive to CAP treatment. Consistent with the findings in HNSCC cells, treatment using CAP resulted in increased apoptosis of HN9, which is a cell line established from an undifferentiated cancer of the parotid gland (28). However, no studies have been conducted to investigate the specific mechanism of the above results. It is interesting to see whether CAP’s anti-neoplastic effect on parotid tumor cells shares a similar mechanism as previously mentioned in CAP’s treatment in head and neck upper ADT SCC (section 2).

## 5 CAP’s treatment for head and neck skin cancers

Common head and neck skin cancers include basal cell carcinoma (BCC), SCC, and malignant melanoma, in decreasing order of disease prevalence (78). One way to explore CAP’s treatment for head and neck skin SCC might be to draw on the experience of successful application of CAP on upper ADT SCC (section 2), as they share histological commonalities. Between melanoma and BCC, the former attracts more attention than the latter.

### 5.1 Malignant melanoma

Melanoma is a malignant tumor that originates from skin melanocytes. Melanoma accounts for only 3% of the entire skin cancer but is responsible for 65% of the skin cancer-related deaths (79). This is mainly because melanoma is more likely to metastasize early with a higher tumor staging, compared to other types of skin cancers. In the advanced stage of melanomas, surgery and chemotherapy provide limited efficacy, leading to a poor five-year survival rate (80). In addition, resistance to chemotherapy is also a challenging problem in the clinical management of melanoma (81). Fortunately, CAP has proven a versatile tool in effectively treating melanoma.

#### 5.1.1 CAP selectively kills melanoma cells

Previous studies have shown that different types of tumor cells have different sensitivity to RONS, leading to treatment sensitivity. Unlike pancreatic cancer cells, melanoma cell lines, such as SK-MEL-28, A375, MaMel86a, and 501-MEL, are sensitive to CAP treatment (82). Interestingly, the cystine-glutamate antiporter xCT (SLC7A11) gene is upregulated in melanoma cell lines which are resistant to CAP treatment. Therefore, we can use SLC7A11 as a screening marker before CAP treatment, aiming to evaluate whether the patient is suitable for CAP treatment.

#### 5.1.2 Managing melanoma using direct CAP treatment

CAP’s versatility in melanoma treatment is manifested in three aspects: direct exposure, indirect treatment, and collaboration with other oncotherapies. The molecular mechanism of each mode will be discussed below. When CAP is applied directly, low doses of plasma could induce senescence in melanoma cells, whereas high doses lead to DNA damage in tumor cells, thus promoting the induction of the sub-G1 phase and increasing apoptosis (83). In another study, it found that cell surface receptor Fas, which is related to the extrinsic apoptosis pathway, participated in the CAP-induced apoptosis in melanoma cell lines (44). This process was triggered by Sestrin2 which was manipulated by CAP. The overexpression of Sestrin2 activated the MAPK signaling pathway, and subsequently increased the level of Fas and Fas ligand. In addition to cancer cell apoptosis, cell migration and adhesion in the melanoma cell lines can also be suppressed by CAP (45). Inhibition of tumor metastasis continues to be one of the main therapeutic objectives in oncology. In this study, CAP reduced tumor cell motility and colony formation without significantly affecting cell metabolism and cell cycle progression.

Further to the above *in vitro* results, CAP’s anti-neoplastic effect was validated *in vivo*. After melanoma was induced in nude mice, the tumor volume was significantly decreased in CAP-treated groups in a dose-dependent manner (84). Most previous cancer studies have focused on the role of ROS as the main physicochemical component of CAP. However, it is well known that CAP contains far more biologically active ingredients than ROS. A genome-wide analysis found that non-ROS constituents were also responsible for the inhibition of tumor cell growth by regulating PTGER3 and HSPA6 (85). Therefore, we suspect that the anti-melanoma effect of CAP is the collective result of various components of plasma.

#### 5.1.3 Managing melanoma using indirect CAP treatment

In addition to direct CAP irradiation, indirect CAP treatment in the form of PAM also demonstrates comparable anti-tumor efficacy in melanoma cells. RONS are described as the primary components accounting for the anti-tumor effect of CAP, and rich and long-lived RONS have been found in PAM (86). On one hand, the modification of amino acids by RONS in PAM may lead to protein inactivation or activation, hence triggering anti-tumor signaling pathways (46). On the other hand, CAP could cause acidification of water and media. Meanwhile, the Ca^2+^ influx induced by CAP in melanoma cells was more prominent in acidic conditions than in physiological conditions. Moreover, CAP induced NO production which caused protein nitrification in melanoma cells under CAP-induced acidic condition (43). In summary, although direct CAP treatment is more efficient, especially for superficial cancer, PAM still has unique advantages. For example, PAM can eliminate deep space tumors and locoregional metastasis by injection, which are not normally accessible by direct CAP irradiation.

#### 5.1.4 CAP combined with other oncotherapy for melanoma

Combining CAP with existing anti-cancer therapies is also a potential treatment option for melanoma. In Alimohammadi’s study, it was found that CAP induced higher lipid peroxidation and NO production in B16 melanoma cells than in normal cells. In addition, CAP combined with chemotherapy (dacarbazine) lead to improved tumor control in mice with melanoma (42). In a parallel study, Daeschlein et al. found that CAP treatment alone showed a significantly delayed tumor growth, although less effective than electrochemotherapy (ECT). But when CAP was combined with ECT, they improved the survival of mice while reducing the dosage of ECT (87). Therefore, this type of joint therapy offers an alternative for patients who cannot tolerate ECT alone.

As previously mentioned, synergy existed between CAP and nanomedicine in HNSCC treatment (section 2). This synergy also applies to melanoma treatment. In Adhikari’s study, CAP combined with silymarin nanoemulsion (SN) destroyed mitochondrial membrane integrity and reduced ATP production in melanoma cells (41). Moreover, the downregulation of the PI3K/AKT/mTOR survival pathway and RAS/MEK transcriptional pathway were also observed in the co-treatment group. These data confirmed that CAP and SN together could activate autophagy in melanoma cells. Further study by the same group discovered that CAP and SN synergistically inhibit human melanoma tumorigenesis by blocking the HGF/c-MET downstream pathway (40).

### 5.2 Basal cell carcinoma

BCC is a slow-growing, locally aggressive malignant epidermal skin tumor with a high incidence in Caucasians (88). Ultraviolet radiation exposure is generally considered to be the main cause of BCC. BCC occurs mainly in the body areas which are exposed to sunlight, most commonly in the head and neck (80% of all cases) (89). Unlike the large number of studies devoted to CAP treatment for melanoma, the number of experiments on BCC treatment using CAP is very limited. In one study by Yang et al., PAM was produced by CAP irradiation of Dulbecco’s modified eagle medium (DMEM) and phosphate-buffered saline (PBS) and used on BCC cells (47). The TE354T BCC cells were cultured with PAM and showed decreased viability and increased apoptosis. Further investigations identified changes in the MAPK and TNF signaling pathways which contributed to PAM-induced apoptosis in BCC cells.

## 6 Recent preclinical and clinical trials in CAP oncology

A series of clinical trials have revealed that CAP has tremendous therapeutic potential for HNCs. This is partially because of CAP’s distinct technical advantages. Some of the most common cancers, such as those arising from solid organs (lung and liver) and gastrointestinal tracts (colorectum and stomach), are anatomically deep. This poses real challenges for direct CAP treatment. In comparison, most cancers in the head and neck should be accessible by CAP, as they are either superficial (skin cancers), subcutaneous (thyroid and parotid glands), or reachable through upper ADT (oral cavity, pharynx, and larynx). Nowadays, surgery is still the mainstay treatment modality for many HNCs. Fortunately, CAP serves as a versatile tool throughout the entire peri-operative period. However, due to the diversity in tumor type, location, and stage, the optimal timing of CAP’s participation varies.

### 6.1 CAP is applied as a preoperative sensitizer

CAP can be used in HNC treatment before surgical resection. Preoperative CAP treatment can induce apoptosis of tumor cells, reduce tumor volume, and decrease the difficulty of surgery. Schuster et al. devised an ingenious experiment in which nine patients with advanced HNC were treated with CAP first, followed by radical surgical resection two weeks later (49). The pathological analysis showed that apoptotic cells appeared more frequently in CAP-treated tissue than in untreated areas. This is coupled with clinically visible tumor surface response. Another clinical study exposed human cutaneous melanoma tissue to CAP (53). The authors identified an increased level of apoptosis and alleviated inflammatory response after CAP treatment.

### 6.2 CAP is applied as an intraoperative assistant

CAP can potentially be used during surgical operations for HNCs as a future-proof standard. Surgeons could use plasma jet intuitively like a hand-held instrument, either in a stand-alone fashion or attached to any common surgical equipment, e.g., scalpel or monopolar diathermy. For surgically challenging tumors, such as cancer near vital structures, e.g., carotid artery, vagus nerve, skull bases and meninges, CAP can be applied intraoperatively owing to its non-thermal nature. This not only avoids damaging the above tissues but also induces cancer cell apoptosis. Hasse et al. treated tissue samples surgically obtained from patients with stage T4 HNC (52). CAP treatment of tumor tissue induced more apoptotic cells than in healthy tissues that were accompanied by elevated extracellular cytochrome C levels.

### 6.3 CAP is applied as a postoperative adjunct

CAP can be used after surgical resection of HNC. Cancer-related symptoms or treatment-associated complications can still be debilitating even after extensive management, especially in palliative HNC patients. CAP is bactericidal and can reduce microbial contamination in heavily infected tumor ulcers, which is often seen in patients with advanced HNC (49). In addition, CAP can promote microcirculation and further facilitate the healing of chronic cancer ulcers (50). Metelmann et al. noticed that most patients afflicted with advanced HNSCC reported a decreased need for analgesics, reduction of typical fetid odor, and wound healing of infected ulcerations after CAP treatment (48). Their subsequent cohort reported that CAP-treated patients with locally advanced (T4) HNC received not only the aforementioned benefits but also improved social function and positive emotional affect (51).

## 7 Limitations of CAP treatment

Although CAP has proven to be a promising head and neck treatment as discussed above, there are also limitations to its application due to its safety profile. Due to the complexity of CAP components, overtreatment with plasma is likely to cause undesired side effects.

For example, the formation of free radicals by plasma raises a concern regarding its potential side effects on HNC patients. In a retrospective analysis of 20 patients suffering from locally advanced HNC who received palliative CAP treatment, there were no severe side effects observed, certainly no life-threatening ones (90). The reported issues were mostly mild reactions, uneasiness, and discomfort, which were tolerated by most patients. These include bad taste, exhaustion, collateral edema, and minimal hemorrhage (51).

Technological limitation also hindered the application of CAP equipment in the clinical treatment of otolaryngology head and neck surgery. Although many portable CAP generators have been developed, they are still unable to be carried into the existing surgical instrument. Due to the special anatomical structure of the head and neck, CAP will have greater potential for clinical application if it can be combined with existing therapeutic instruments such as nasal endoscopy.

## 8 Conclusion

Plasma oncology using CAP has been gaining increasing attention in HNC treatment, owing to CAP’s several advantages. Firstly, CAP is a comprehensive therapy covering the entire disease spectrum of HNC, including upper ADT cancers, glandular cancers, and head and neck skin cancers. Secondly, CAP demonstrates excellent technical versatility, as it can be used either directly as superficial irradiation or indirectly in the form of PAM, each with distinct advantages. Thirdly, CAP serves as a multimodal treatment for HNCs, as it can not only work independently but also collaborate with other existing oncotherapies, such as chemotherapy, targeted therapy, and immunotherapy. Microscopically, CAP affects various cellular events, such as cell apoptosis and cell invasion, through several potential molecular mechanisms, such as the p53 and MAPK signaling pathways. Macroscopically, CAP has manifested great potential in recent clinical trials, which is best exemplified by its participation during the entire perioperative period and management of post-treatment cancer-related complications. Fortunately, CAP oncotherapy did not excel at the price of causing severe side effects to the HNC patients. It is still considered a safe and patient-friendly treatment modality. Overall, CAP oncotherapy has significantly strengthened the arsenal for fighting HNCs.

## Author contributions

FT: Conceptualization, methodology, validation, formal analysis, resources, data curation, writing, supervision, project administration, funding acquisition. XL: Formal analysis, resources, data curation, writing, visualization. XR: conceptualization, methodology, formal analysis, resources. DL: Software, resources, visualization. YW: Resources, supervision. All authors contributed to the article and approved the submitted version.

## Funding

This work is sponsored by the Fundamental Research Funds for the Central Universities and Tongji University Affiliated Shanghai Fourth People's Hospital Startup Research Funding.

## Acknowledgments

The authors wish to thank Relyon Plasma GmbH (Regensburg, Germany) for providing the handheld CAP device, Piezobrush PZ2, used **Figure 1**. **Figure 2** was created with BioRender.com.

## Conflict of interest

The authors declare that the research was conducted in the absence of any commercial or financial relationships that could be construed as a potential conflict of interest.

## Publisher’s note

All claims expressed in this article are solely those of the authors and do not necessarily represent those of their affiliated organizations, or those of the publisher, the editors and the reviewers. Any product that may be evaluated in this article, or claim that may be made by its manufacturer, is not guaranteed or endorsed by the publisher.
